# Addressing Research Readiness Challenges in African Institutions: Development and Application of a Modular Assessment Tool

**DOI:** 10.12688/gatesopenres.16368.1

**Published:** 2025-10-27

**Authors:** Patrick Amboka, H Kariuki, Nosa Orobaton, Alphonsus Neba, Julius Kirimi Sindi

**Affiliations:** 1 African Population and Health Research Center, Nairobi, Nairobi County, Kenya; 2 Bill & Melinda Gates Foundation, Seattle, Washington, USA

**Keywords:** Institutional capacity, Research capacity, Research readiness assessment, Capacity gaps, Capacity strength, African institutions, Institutional research areas, Institutional performance

## Abstract

**Introduction:**

Historically, African research institutions have faced significant barriers to gaining recognition on a global stage due to limited infrastructure, underdeveloped governance frameworks, and low representation in high-impact publications. This underrepresentation reflects systemic barriers such as the lack of visibility of both researchers and institutions, limited funding, inadequate infrastructure, and fragmented institutional arrangements, which impede the continent’s ability to contribute robustly to the global knowledge economy. To address these barriers, the Research Readiness Assessment Survey (RRAS) was developed as a modular, context-specific tool to evaluate and enhance institutional research capacity across multiple dimensions, including research infrastructure, policy and policy engagement, governance, human resources, institutional arrangements, grant management, and research outputs.

**Methods:**

The RRA was developed by integrating different global frameworks. This assessment adopted a cross-sectional design. Piloting was performed in nine institutions distributed across Kenya, Ethiopia, and Nigeria. The questionnaire used for data collection was uploaded to RedCap in English, French, and Portuguese. The tool underwent validity and reliability testing. Validity testing included piloting of nine institutions. Data from the pilot test were categorized and analyzed using STATA version 17.0. Analyses were performed at both the univariate and bivariate levels.

**Results:**

The proportion of institutions that performed, on average, in all modules, was 66.67% (n=6). None of the institutions had a strong overall institutional performance based on our scoring. The proportion of institutions that had developed overall institutional performance was 22.22% (n=2), whereas 11.11% (n=1) had limited overall institutional performance. There was a statistically significant and strong positive correlation between the following module scores and overall institutional performance: laboratory infrastructure, institutional arrangement, grant management, policy and policy engagement, project management, and human resources; [r=0.666; p-value<0.05],[r=0.916; p-value<0.001], [r=0.799; p-value<0.01], [r=0.660; p-value<0.05], [r=0.738; p-value<0.05], and [r=0.648; p <0.05], respectively.

## Introduction

Africa boasts many institutions and organizations engaged in research across diverse disciplines such as health, agriculture, social sciences, and environmental sciences. ^1^ However, despite this extensive network, Africa’s contribution to global research remains disproportionately low, accounting for only 1–2% of the global scientific output. ^2^ This underrepresentation reflects systemic barriers such as the lack of visibility of both researchers and institutions, limited funding, inadequate infrastructure, and fragmented institutional arrangements, which impede the continent’s ability to contribute robustly to global knowledge. ^3, 4^ As documented in previous studies, these challenges have perpetuated inequities in resource allocation and collaboration opportunities. ^2, 5^

The opacity of African institutions online complicates the efforts of the collaborators, funders, and policymakers to engage with these institutions. Therefore, it is common to find that donors often favor a small group of well-known universities and researchers from selected countries who dominate partnerships and funding allocations. ^6^ Further, international support to Africa’s research systems has traditionally prioritized technical assistance initiatives. For example, low- and middle-income countries (LMICs) received only 0.2% of more than 69,000 biomedical grants listed on World RePORT in 2016. ^7^ Additionally, investments in African research institutions should be long-term and strategic and institutions should be able to manage funds effectively. ^8^ Most interventions conducted since the turn of the century have generally failed to create a critical mass of well-trained and networked researchers across the continent, increasing research productivity, supporting university-wide systems critical to success and sustainability in research and training in Africa, address issues related to inadequate local training and poor retention of human resources for research, research leadership, and information access, and strengthen the interfaces between research producers and users. This, in short, has led to the scarcity of centers of research excellence on the continent and continued dependence on the north. ^9^ Most African research involves collaborations between countries and institutions from the global north and south. ^10^

Institutional readiness, encompassing governance, infrastructure, and human resources, is a critical determinant of an institution’s ability to produce high-quality research and forge international collaboration. However, assessing readiness remains a challenge in Africa, largely because of fragmented and incomplete institutional capacity datasets. ^4^ Insufficient national research frameworks and policies affect African research systems. Institutions are characterized by critical gaps in infrastructure and human resources. ^11^ Systemic inequalities are a major challenge for national policies and research frameworks within African research institutions. Countries such as Kenya and South Africa have made strides; however, many African nations continue to struggle with systemic inequalities in research capacity and output. ^11^

The Research Readiness Assessment Survey (RRAS) addresses these challenges by systematically capturing and quantifying data on research readiness across thematic areas such as infrastructure, institutional arrangements, policy and policy engagements, collaborations, research outputs, and human resources. It was designed specifically for the African context, which evaluates institutions across multiple dimensions, including human resources, infrastructure, institutional arrangements, collaborations, policy and policy engagements, grant management, project management, institutional experience, international certifications, research outputs, and institutional and staff recognition. By offering standardized metrics, the assessment facilitates evidence-based decision making for funding allocation, thus bridging critical data gaps. ^11^ The tool will also contribute to the global discourse on strengthening research capacity by providing empirical insights into the development and application of context-specific tools for resource-constrained settings.

Research conducted in Africa operates across differentials in power, knowledge, and resources. ^10^ However, equitable international research collaborations utilizing local expertise can level this difference. ^12^ In addition, effective institutional arrangements can improve policy integration. ^13^ Addressing these challenges is imperative, not only to enhance Africa’s integration into the global research landscape, but also to tackle pressing regional issues such as public health, climate change, and food security. ^3^ Initiatives such as the African Research Universities Alliance (ARUA) and Africa Research Connect (ARC) represent steps in the right direction but underscore the need for standardized tools to systematically assess and enhance institutional capacities across the continent. ^4, 11^

## Methodology

### Research Readiness Assessment (RRA) Tool Development Process

The RRA tool development followed a structured, multi-phase process to generate localized, relevant, and inclusive assessment components.

### Phase 1: Integration of Global Frameworks and Standards

The development of the survey tool was guided by a comprehensive conceptual framework that integrated global best practices with the unique challenges and opportunities of African research institutions. This framework emerged from an exhaustive review of international competency frameworks, standards, and tools, ensuring that the tool is both rigorous and contextually relevant.

The first phase involved an in-depth analysis of global tools and standards to identify the key metrics for assessing institutional readiness. These frameworks and standards were critically evaluated to adapt their methodologies and insights to the African research context. The frameworks and tools reviewed include the following.

### Global Health Research Competencies

The Global Health Research and Development Competencies Checklist provides a foundation for evaluating institutional capacity for multidisciplinary health research. Its emphasis on cross-sectoral collaboration and innovation has informed the development of metrics addressing institutional readiness for complex research environments.

### Good Financial Grants Practice (GFGP)

GFGP standards, widely regarded as the gold standard for research institutions’ financial governance, contributed to essential metrics for assessing an institution’s capability to manage research funding effectively and transparently. ^4^

### Clinical Research Competency Frameworks

The TDR Global Competency Framework for Clinical Research is instrumental in understanding the skills, organizational structures, and operational requirements for conducting clinical research, particularly in resource-constrained settings. This framework emphasizes the importance of building institutional capacity to manage clinical trials and adhere to international standards.

### Global Health Investment Competencies

The Bill & Melinda Gates Foundation competency requirements highlight the critical attributes of institutions involved in health-focused research investments. These requirements helped align the tool with the expectations of major global funders, ensuring that African research institutions could meet the international benchmarks for research quality and governance.

### Education Sector Competency Standards

Insights from the education project standard tools informed the inclusion of metrics for evaluating governance, stakeholder engagement, and research management in education-focused institutions. This allowed the tool to address critical gaps in the assessment of readiness for education-related research initiatives.

### International Organization for Standardization

A review by the International Organization for Standardization for data quality and governance ensured that the tool met internationally recognized benchmarks for data collection, management, and reporting. These standards enhance the tool’s credibility and robustness in evaluating institutional capacity.

### Phase 2: Stakeholder Consultations

Extensive consultations with African researchers, institutional administrators, funders, and policymakers were conducted to contextualize the findings from tool development. The consultations with the stakeholders were conducted through both physical and virtual meetings. The stakeholders represented different types of research institutions (Public, private), different countries (Anglophone, Francophone, Lusophone), and different levels of experience (Experienced researchers and early career researchers). Stakeholders emphasized the need for metrics that accounted for diverse linguistic and cultural contexts. The stakeholder consultation meeting was attended by 15 experienced researchers holding leadership positions within their institutions representing different country language groups as well as the type of institution. The meeting was also attended by 10 early career researchers representing different country language groups and the type of institutions as well.

### Phase 3: Piloting of the RRAS

To test for validity and reliability, the tool was piloted in three countries: Kenya, Ethiopia, and Nigeria.

**Validity**

Piloting and tool validation were performed to check the practical usability, scientific accuracy, and reliability of the tool. The tool underwent further modifications based on feedback from piloting and validation exercises. The tool was reviewed with stakeholders from Kenya, Ethiopia, Nigeria, and Mozambique, and customized to fit different contexts. Some health questions have also been validated using organizational readiness tools for global health intervention. ^14^

Key learnings from the piloting and validation exercises led to the integration of a modular approach for different sections, automated reminders, error tracking, and progress monitoring features, enhancing the tool’s usability and accuracy. The tool can also be applied to the whole institution or part of the institution, such as a school of medicine or public health, where the whole institution is not ready to conduct the survey. To ensure data integrity, the tool employed logic checks, predefined response categories, and automated error notifications. These measures facilitated the collection of high-quality standardized data.

**Reliability**

The tool was filled out by a subgroup of participants on two separate occasions to check for consistency in the results. A high correlation between the scores was an indicator of good reliability. As multiple assessors were involved, all measures were put in place to ensure agreement in scoring and interpretation. Reliability checks were conducted by having different assessors from the same institution independently rate it and then calculate interrater agreement. To minimize variability, clear instructions were included on the first page of the tool. This was performed to ensure that accurate and complete data were collected. The researchers implemented data quality checks when the tool was being filled, to promptly identify and correct errors.

### Assessment design

This study used a cross-sectional design. It entailed quantitative data collection with both open- and closed-end questions.

### Assessment setting

Pilot testing and tool validation were conducted in nine institutions across Kenya, Ethiopia, and Nigeria. The pilot institutions were selected through random sampling. Universities were targeted because it is assumed that almost all universities in Africa carry out research (although insufficient), apart from focusing on academics. ^15^

### Inclusion criteria

1. African research institutions of higher education
2. African research institutions
3. Government departments
4. Research groups and units

### Exclusion criteria

1. Non-African research institutions
2. Institutions that are no longer involved in active research

### Assessment instruments

Structured and unstructured questionnaires were used for assessment. The assessment was uploaded to the RedCap platform to ensure accuracy, validity, and completeness. ^16^ The tool was developed in English and was translated into French and Portuguese. The tool was then shared across African research institutions by in-country collaborators and 12 Kenyan research assistants. In-country collaborators and research assistants underwent through training prior to circulating the survey link.

### Survey distribution procedure

Several databases were used to list over 7,000 African research institutions. Some of the databases utilized include ARC, the Association of African Universities (AAU), ARUA, and regional African bodies such as the Committee of Vice-Chancellors of Nigerian Universities (CVCNU), and the Commission of Universities Education, Kenya, among others. African research institutions were also listed by the Commission of higher education in different African countries. The team was able to access and validate the contact information from a significant number of African universities and research institutions. Contact information (email address and/or phone number) was extracted from institutions’ websites, ARC, journal databases, networks such as Author AID, institutional contact details from in-country collaborators, and referrals from other institutions.

The implementation of RRA distribution involved a carefully structured, phased rollout across institutions to ensure maximum coverage, data accuracy, and institutional engagement. With the help of in-country collaborators and Research Assistants (RAs) based in Kenya, we contacted institutions and shared with them a data capture survey to nominate the relevant person to complete the assessment. The initial contact people were the research directors and the vice-chancellors/their equivalent, or any person from the institution, where we couldn’t retrieve the research directors' and the vice-chancellors' contacts. We first contacted the research directors and vice-chancellors/their equivalent because they can give reliable information by themselves or through their nominees, and their contact information is usually available online and through publications where they are corresponding authors. The assessment was then shared with the nominee through the RedCap platform for ease of tracking responses and progress. Once the nominee received the survey, it was his/her responsibility to either populate the whole survey (If they had all the information) or share with the different departments to populate their sections. The assessment link was also shared with some institutions via email. The RAs were engaged in communicating directly with institutions via email and phone calls. These communications emphasize the importance of completing the assessments.

### Collaborations and sensitization

To promote awareness and participation, the team collaborated with key African research networks and organizations. These partners provided crucial support for identifying and sensitizing institutions across the continent regarding the importance of completing the assessment. Through these collaborations, the team was able to build a comprehensive list of African research institutions, significantly expanding its assessment reach.

### Technical support

A dedicated support team was established to address technical issues encountered by the institutions during the data collection process. This team handled challenges, such as server downtimes and data inconsistencies. In addition, real-time troubleshooting support was provided to ensure a seamless user experience.

### Data analysis

The validation data were analyzed using STATA version 17.0. Data cleaning was performed using frequency distribution. Analyses were performed at both the univariate and bivariate levels. Descriptive statistics were conducted for the continuous variables, and the data were described using means and medians. The variables were classified into 11 modules: institutional research infrastructure (laboratories and libraries), institutional experience, institutional arrangement, grant management, policy and policy engagement, project management, institution and staff recognition, collaborations, international certifications, and human resources. The details of each module are listed in Table 1.

**Table 1.** Example of assessment areas in each module.

Module	Examples of assessment areas
Research Infrastructure	• Data on laboratory facilities, equipment, and biosafety classifications.
Institutional Experience	• Opportunities for Earlier Career Researchers. • Opportunities for minority groups. • Presence of an electronic management system for data archiving. • Academic journals hosted by the institution.
Institutional Arrangement	• The presence of visible standards Operating procedures (SOPs) and frequency of updating. • Process of expediting procurement during emergencies. • Scholarships for postgraduate students. • Outreach efforts include admission policies that encourage minority groups. • Presence of a gender policy. • Presence of an institutional ethics review committee.
Grants Management	• Management of donor grants. • Establishment of an electronic grants management system within the institution. • Capacity to compete and deliver work on large international grants. • The presence of a dedicated research grants management office.
Policy & Policy Engagement	• The presence of a person responsible for ensuring research uptake by policymakers. • Strategies used to engage policymakers.
Project Management	• Having a dedicated project manager, their certification, and years of experience.
Institution and Staff Recognition	• Awards and recognition by the institution and employees in the past 3 years.
Collaborations	• Number of collaborations in the past 3 years.
International Certifications	• Number of international certifications the institution holds.
Human Resource	• Presence of a principal investigator (s) and their qualifications. • Levels of staff.
Research Outputs	• Number of peer-reviewed publications.

Each module was then classified as leading edge (91-100%), Strong (70-90%), Average (50-69%), Developing (30-49%), and limited (below 30%). Correlations between the different modules and overall scores were determined. Linear regression was used to predict the outcomes (overall scores). The results were disaggregated by continent, region in Africa, and country.

The performance of each module was calculated by obtaining the percentage based on the total number of possible points. The expected maximum score for every institution was 130 points. The maximum points per module are presented in Table 2.

**Table 2.** Maximum points for each module.

Module	Maximum points
Research Infrastructure	28
Institutional Experience	14
Institutional Arrangement	18
Grants Management	9
Policy & Policy Engagement	14
Project Management	7
Institution and Staff Recognition	8
Collaborations	8
International Certifications	3
Human Resource	16
Research Outputs	5
Total	130

## Results

Tool piloting and validation were performed at nine institutions across three countries (Kenya, Ethiopia, and Nigeria), as shown in Table 3.

**Table 3.** Distribution of institutions used in data validation.

Country	Number of institutions
Kenya	4
Ethiopia	4
Nigeria	1
Total	9

### Infrastructure capacity

Analysis of infrastructure capacity revealed significant variability in the availability of central health research and development laboratories and libraries that able to support health research. On average, the institutions reported having seven laboratories, with a median of 5. This distribution was marked by a wide range, from 0 to 67 laboratories. The standard deviation of 10 further underscores this variability, pointing to substantial differences between institutions with minimal and extensive laboratory facilities. Similarly, the availability of libraries able to support health research showed an average of 3 libraries per institution, with a median of 2. The range spanned from libraries 0 to 17, reflecting moderate variability, as indicated by a standard deviation of 3.2, as shown in Table 4.

**Table 4.** Summary of Institutional Infrastructure Capacity.

	Mean	Median	Min	Max	Std Dev	Range
Number of Laboratories	6.9	5.0	0	67.0	10.0	67.0
Number of Libraries	3.0	2.0	0	17.0	3.2	17.0

### Research outputs

The analysis focuses on peer-reviewed journal articles. Research output varied widely across institutions, reflecting disparities in research output and readiness for dissemination. On average, institutions produce 145 peer-reviewed publications annually. This skewed distribution was further highlighted by the range that spanned from 0 to 2,000 publications, as shown in Table 5. The dominance of a small number of high-output institutions contributed to the high standard deviation of 276.1, indicating significant variability in research productivity. The Publications Readiness Score, an indicator of institutions’ capacity to disseminate research effectively, averaged 0.75, suggesting moderate overall readiness.

**Table 5.** Summary of Institutional Research Outputs.

	Mean	Median	Min	Max	Std Dev	Range
Peer-Reviewed Publications	144.9	52.0	0	2000.0	276.1	2000.0

### Institutional research staff

Research staffing level has emerged as a critical determinant of institutional readiness for research. On average, the institutions reported seven research staff members, with a median of six, as shown in Table 6. The range of staff levels varied from 1 to 20, indicating that some institutions operate with very limited personnel, whereas others have more robust research staffing levels. A standard deviation of 2.8 reflects moderate variability in this metric. The staff readiness score, which captures metrics related to research staffing quality and capacity, averaged 0.62, indicating an average performance across institutions. These results emphasize the need for targeted capacity-building initiatives to address research staffing gaps and enhance human resource readiness.

**Table 6.** Summary of Institutional Research Staff.

	Mean	Median	Min	Max	Std Dev	Range
Research Staff	6.9	6.0	1	20.0	2.8	19.0

### Overall Institutional Performance in each Module

The proportion of institutions that performed, on average, in all modules was 66.67% (n=6). None of the institutions were leading edges or as strong as overall institutional performance. The proportion of institutions that had developed overall institutional performance was 22.22% (n=2), whereas 11.11% (n=1) had limited overall institutional performance. Regarding institutional research infrastructure (laboratories and libraries), the proportion of institutions that were leading edge was 33.33% (n=3); 11.11% (n=1) had strong performance, and the same proportion had developing performance. The proportion of institutions with limited performance was 33.33% (n=3). In terms of institution and staff recognition, all institutions assessed had limited performance (100%, N=9). All institutions assessed on research outputs (peer-reviewed publications) were either leading edge, strong, or average: 55.56% (n=5), 22.22% (n=2), and 22.22% (n=2), respectively. The assessment of institutional experience was either average, developing, or limited, at 22.22% (n=2), 66.67% (n=6), and 11.11% (n=1), respectively, as shown in Table 7.

**Table 7.** Overall Institutional Performance in each Module.

	Leading edge	Strong	Average	Developing	Limited
Institution Research Infrastructure (Laboratories and Libraries)	33.33% (n=3)	11.11% (n=1)	11.11% (n=1)	11.11% (n=1)	33.33% (n=3)
Institutional Experience	0% (n=0)	0% (n=0)	22.22% (n=2)	66.67% (n=6)	11.11% (n=1)
Institutional Arrangement	0.00% (n=0)	22.22% (n=2)	33.33% (n=3)	33.33% (n=3)	11.11% (n=1)
Grants Management	0.00% (n=0)	44.44% (n=4)	33.33% (n=3)	11.11% (n=1)	11.11% (n=1)
Policy & Policy Engagement	0.00% (n=0)	11.11% (n=1)	44.44% (n=4)	22.22% (n=2)	22.22% (n=2)
Project Management	0.00% (n=0)	33.33% (n=3)	11.11% (n=1)	0.00% (n=0)	55.56% (n=5)
Institution and Staff Recognition	0.00% (n=0)	0.00% (n=0)	0.00% (n=0)	0.00% (n=0)	100.00% (n=9)
Collaborations	33.33% (n=3)	44.44% (n=4)	0.00% (n=0)	11.11% (n=1)	11.11% (n=1)
International Certifications	11.11% (n=1)	0.00% (n=0)	0.00% (n=0)	88.89% (n=8)	0.00% (n=0)
Research Staff	0.00% (n=0)	22.22% (n=2)	44.44% (n=4)	22.22% (n=2)	11.11% (n=1)
Research Outputs	55.56% (n=5)	22.22% (n=2)	22.22% (n=2)	0.00% (n=0)	0.00% (n=0)
**OVERALL SCORE**	**0.00% (n=0)**	**0.00% (n=0)**	**66.67% (n=6)**	**22.22% (n=2)**	**11.11% (n=1)**

## Feedback from data validation

### Average length of assessment

The average time taken to complete the assessment by the institutions was 12 hours. All the assessments were completed by the research directors.

### More readily accessible data sources

The more readily accessible data sources included data on institutional infrastructure, international certifications, institutional research areas, human resources, project management, and staff recognition. The people filling in the assessment were able to get the data without consulting and liaising with other departments within their institutions.

### Less readily accessible data sources

The less readily accessible data sources included institutional research outputs, institutional experience, data on grants received, institutional arrangements, policy engagements, and data on collaborations. The less readily accessible data forced the data collator to liaise with other departments to get comprehensive data.

### Clarity of the RRA

The validation exercise showed that the questions in the assessment were clear, and those that were not clear were corrected. Adequate information and guidance were also included at the beginning of each section since the tool is self-administered.

### Respondent burden

It was reported from the data validation exercise that the assessment was time-consuming, and there should be clear communication with the target institution on the length of the assessment prior to starting it. “Auto save” and “save and return later” functionalities were included in the assessment to prevent data loss and for convenience, respectively.

### Perceived value as reported by Pilot Institutions

1. Holistic diagnostic of research capacity: RRA offers a comprehensive, modular view of institutional research readiness, covering governance, infrastructure, human capital, research support services, and inclusivity. This broad lens allows institutions to identify strengths and pinpoint systemic gaps that might go unnoticed.
2. Evidence-based planning and benchmarking: Institutions can use RRA results to prioritize investments and monitor progress over time, enabling strategic planning and internal benchmarking against peers or prior performance.
3. Engagement and ownership: The participatory nature of the assessment process fosters internal stakeholder engagement, encouraging departments and leadership to take ownership of capacity-strengthening plans.

### Lessons learned from the authors perspectives

1. Self-reporting requires supplementary validation: Reliance on self-reported data poses risks of bias or inconsistency. Future improvements to the tool should integrate independent validation mechanisms to strengthen data quality and credibility.
2. Digital tools can enhance institutional engagement: The modular design, multilingual interface, and automation features of the RRA (e.g., reminders, logic checks) were instrumental in improving data quality and participation, proving the value of well-designed digital platforms in institutional assessments.
3. Regional inequities require tailored outreach and support: Francophone and Lusophone institutions were underrepresented in early data collection, highlighting the need for linguistically and culturally responsive engagement strategies.
4. Stakeholder engagement is crucial for tool relevance and adoption: Early and continuous input from program officers, administrators, and funders enriched the tool’s design and applicability, reinforcing the importance of co-creation in tool development.

## Discussion

The results of this assessment highlight key institutional research capacity gaps and strengths within the piloted institutions. The assessment uncovered systemic gaps in infrastructure, human resources, grant management, policy engagement, and project management across African research institutions, providing critical insights that align with and expand upon the existing literature. This discussion contextualizes the findings within the broader body of research and examines their implications for policy and practice, while identifying areas for future exploration.

The analysis revealed significant disparities in infrastructure readiness across institutions, with some reporting well-developed central health research and development laboratory and library facilities able to support health research, while others operated minimally to not. These findings are consistent with those of a previous study that uncovered an uneven distribution of research infrastructure across African research institutions. ^2^ The limited facilities in many African research institutions hamper their ability to conduct high-quality research and attract funding. ^6^ The assessment of the infrastructural capacity can have some limitations as some institutions could have more numbers but lesser capacity.

The analysis revealed disparities in peer-reviewed publications, with a small subset of institutions accounting for most peer-reviewed publications. This information may have limitations as the lead authors of the publications may be affiliated in non-African research institutions whereas the assessment only looks at the number and not the roles in the publications.

Institutional arrangement is key because it promotes collaboration, leads to improvement in infrastructure, streamlines decision-making, and increases research products. These findings are in line with an earlier study that established that institutional arrangements have a strong influence on the overall performance of an institution. ^17^

We established institutional capacity gaps in institutional grant management among the piloted institutions. Good grant management enhances the timely allocation and release of funds, proper utilization of resources, and compliance with funding requirements. However, poor grant management leads to resource mismanagement, leading to reduced future funding opportunities. Reducing funding opportunities can limit infrastructure development and research productivity within institutions. Grant management has proven to be associated with overall institutional performance. ^18^

A critical gap identified in this assessment was the insufficiency of human resources (research staff) across many institutions. Low research staff levels and lack of diversity in staffing structures have emerged as significant barriers to achieving research readiness. An earlier study emphasized the shortage of skilled researchers and research managers in African research institutions. ^2^ However, the results of this assessment add depth by demonstrating the tangible impact of these shortages on research output and institutional performance.

The assessment established institutional capacity gaps and strengths in policy engagement. Policy engagement is important in evidence-informed decision-making. The results from this assessment aligning with the World Bank’s findings are that there is minimal policy engagement in decision-making in LMIC. ^19^ The assessment established that collaboration among institutions, government agencies, and researchers was a challenge among the piloted institutions. This is an interesting finding since regional collaboration is crucial for bridging disparities between high-performing and low-performing institutions. Collaborations facilitate knowledge transfer, joint research projects, and capacity-building initiatives, and can create opportunities for less-resourced institutions to benefit from the expertise and infrastructure of their more established counterparts. A previous study established that collaborations through regional networks can enhance resource sharing and foster a sense of collective responsibility for advancing Africa’s research agenda. ^20^

The implications of this assessment include infrastructural investment, human resource development, data-driven decision making, improvement in grant management, improvement in project management, and promotion of inclusivity. These implications underscore the interconnected nature of institutional research readiness factors and the need for comprehensive and systemic approaches to capacity-building. By addressing infrastructure gaps, investing in human resources, leveraging data-driven insights, promoting inclusivity, and fostering collaboration, stakeholders can create a more equitable and robust African research ecosystem. This approach not only strengthens individual institutions but also enhances the continent’s collective ability to contribute to global knowledge and innovation.

## Conclusion

In effect, the modular design of the RRA tool increased usability, relevance, and institutional ownership. It lowered the barrier to entry, allowed for customized assessment, and supported meaningful dialogue across departments, all of which contributed to higher engagement and more actionable insights. The findings from this assessment reveal critical areas for targeted interventions to enhance research readiness and capacity. These implications address systemic gaps and offer actionable pathways for stakeholders to strengthen the continental research ecosystem. This assessment represents a significant advancement in addressing the challenges of visibility within Africa’s research ecosystem. By providing a structured and data-driven framework to evaluate institutional capacities, the assessment of transformative impact on the visibility of African research institutions illuminates disparities and provides actionable insights for stakeholders.

## Author contributions

• **Amboka P**: Conceptualization, data curation, formal analysis, investigation, methodology, project administration, software, validation, visualization, and writing – Original Draft Preparation.
• **Kariuki H**: Conceptualization, Data Curation, Investigation, Methodology, Writing – Review & Editing
• **Orobaton N**: Funding Acquisition, Resources, Supervision
• **Neba A**: Funding Acquisition, Investigation, Methodology, Resources, Supervision, Validation, Writing, Review & Editing.
• **Kirimi Sindi J**: Conceptualization, data curation, formal analysis, funding acquisition, investigation, methodology, resources, supervision, writing – Original Draft Preparation, Writing – Review & Editing.

## Ethics and consent

This assessment entailed evaluating systems, infrastructure, policies, and institutional processes rather than personal or sensitive data from human participants. The assessment relied on publicly available information within the institution rather than direct data collection from individuals. The assessment entailed a checklist on publicly available information which include the number of libraries within an institution, and the number of peer reviewed publication the institution has published. The different sections of the assessment were completed by a representative from the institution who had secondary data about the section. For example, modules about the publications were completed using data from the libraries, whereas modules about the number of students were completed using data from the student registrar’s office. However, the assessment adhered to general ethical principles of transparency and no personal identifiers were assessed. The protocol and other assessment documents were approved by the Ethiopian Public Health Association (EPHA) Institutional Review Board (Reference number: EPHA/OG/1006/23). The approval was granted on November 29, 2023. The data collected will be used to generate institutional profiles for public good and therefore the data are not confidential. The institutional profiles will be included on the ARC platform. They will be accessed by funders, institutions, and individuals to identify opportunities in funding and collaboration. The institutional assessment data will not be anonymized so as to achieve the overall goal of improving the visibility of African research institutions through a true representation of their profiles.

## Data Availability

Zenodo: Research Readiness Assessment Validation dataset, Assessment Guide and Scoring Template, https://doi.org/10.5281/zenodo.14614154. ^21^ This project includes the following data:
- Validation dataset associated with this assessment - Assessment guide - Assessment scoring template Data are available under the terms of the Creative Commons Attribution 4.0 International license (CC-BY 4.0).

## References

[1] Nyika J Dinka MO : Integrated approaches to nature-based solutions in Africa: Insights from a bibliometric analysis. *Nature-Based Solut.* 2022;2(April):100031. 10.1016/j.nbsj.2022.100031

[2] Cloete N Schalkwyk F van Winnink J : Researching research universities in Africa. A Research Agenda for Global. *High. Educ.* 2022;131–152. 10.4337/9781800376069.00013

[3] Sawyerr A : 8 - African Universities and the Challenge of Research Capacity Development. *J. High. Educ. Afr.* 2003;2(1):211–240. 10.57054/jhea.v2i1.1687

[4] Fosci M Loffreda L : Strengthening Research Institutions in Africa: A Synthesis Report. 2019; (December). Reference Source

[5] Tijssen R Kraemer-Mbula E : Research excellence in Africa: Policies, perceptions, and performance. *Sci. Public Policy.* 2018;45(3):392–403. 10.1093/scipol/scx074

[6] Salihu ST : Challenges experienced by early career researchers in Africa. *Futur. Sci. OA.* 2020;6(5):3–6. 10.2144/fsoa-2020-0012 PMC7273411 32518684

[7] Ralaidovy AH Adam T Boucher P : Resource allocation for biomedical research: Analysis of investments by major funders. *Heal. Res. Policy Syst.* 2020;18(1):1–9. 10.1186/s12961-020-0532-0 PMC7027210 32066463

[8] Paper P : Recommendations for Strengthening African Research Universities. 2020; (February).

[9] Ezeh AC Izugbara CO Kabiru CW : Building capacity for public and population health research in Africa: The consortium for advanced research training in Africa (CARTA) model. *Glob. Health Action.* 2010;3(1):5693. 10.3402/gha.v3i0.5693 21085517 PMC2982787

[10] Sorensen: LSHTM Research Online. 2017;61:61–67.

[11] Ogega OM Majani M Hendricks C : Research capacity strengthening in Africa: Perspectives from the social sciences, humanities, and arts. *Sci. African.* 2023;20:e01708. 10.1016/j.sciaf.2023.e01708

[12] Rivero C : There’s a science research gap in Africa. Here’s how to fill it. Reference Source

[13] Peters BG : The challenge of policy coordination. *Policy Des. Pract.* 2018;1(1):1–11. 10.1080/25741292.2018.1437946

[14] Dearing JW : Organizational readiness tools for global health intervention: A review. *Front. Public Health.* 2018;6(MAR):1–6. 10.3389/fpubh.2018.00056 29552552 PMC5840160

[15] Njuguna F Itegi F : Research in institutions of higher education in Africa: Challenges and prospects. *Eurasian Multidiscip. Forum.* 2013;1(December):352–361.

[16] Harris PA Taylor R Thielke R : Research electronic data capture (REDCap)-A metadata-driven methodology and workflow process for providing translational research informatics support. *J. Biomed. Inform.* 2009;42(2):377–381. 10.1016/j.jbi.2008.08.010 18929686 PMC2700030

[17] Pohan N : The Effect of Institutional Arrangements and Human Resources Development on Employee Performance in North Sumatra Provincial Suitable Assessment Institutions. 2021.

[18] Dadu TZ Njeri I Kemunto A : Grant management practices and financial sustainability among Non-Governmental Organizations in Taita-Taveta County: A Case of World Vision Kenya. *Int. Acad. J. Econ. Financ.* 2024;4(2):104–140.

[19] World Bank: Improving the quality and quantity of scientific research in Africa. 2019. Reference Source

[20] Vanheukelom J Byiers B Medinilla A : With EU programming for the 2021-2027 Multiannual Financial Framework underway, a new “geopolitical”. *European Rewiring support to African continental and regional organisations.* 2020;268. Reference Source

[21] Amboka P Kariuki H Orobaton N : Research Readiness Assessment Validation dataset, Assessment Guide and Scoring Template. *Zenodo.* 2025. 10.5281/zenodo.14614154

